# Comparative Perfusion Analysis of Free Muscle-Sparing Versus Pedicle Transverse Rectus Abdominis Myocutaneous (TRAM) Flaps *in Vivo* in the Peri-Operative and Late Post-Operative Periods

**Published:** 2017-05

**Authors:** Richard McNally, Jonathan Rimler, Vincent Laurence, Keyianoosh Z. Paydar, Garrett A. Wirth

**Affiliations:** 1 Department of Plastic Surgery, University California Irvine, 200 South Manchester Ave. Suite 650, Orange CA 92868, USA;; 2 Denotes Co-Senior Authorship

**Keywords:** Perfusion, Free muscle-sparing, Pedicle transverse rectus abdominis myocutaneous, Flap, TRAM

## Abstract

**BACKGROUND:**

Current teaching suggests increased perfusion in free transverse rectus abdominis myocutaneous (TRAM) flaps over pedicled TRAM flaps, broadening indications for its use in high risk patients. This study compared perfusion analysis of free muscle-sparing versus pedicle TRAM flaps *in vivo* in the peri-operative and late post-operative periods.

**METHODS:**

The SPY-Elite system using indocyanine green dye was used to analyze flap perfusion intra-operatively and at 1 week and 3 months post-operatively. Image analysis was completed by evaluating the perfusion maps from the SPY- Elite system with Image J software calculate maximum, minimum, and average luminescence over the surface area of the flaps. Student’s T-test was used for statistical analysis.

**RESULTS:**

Intra-operatively, we found a 73.4% greater perfusion in the free muscle-sparing as compared to the pedicled TRAM. This increase in free muscle-sparing TRAM perfusion was not evident 1 week post-operatively, due to a relative increase in pedicle flap perfusion that coincided with a revision of the pedicled flap due to distal flap necrosis. At 3 months, the free muscle-sparing TRAM flap once again showed superior perfusion with a 15.7% increase over the pedicled flap.

**CONCLUSION:**

We showed superior free muscle-sparing TRAM perfusion in the early peri-operative period which coincided with the time framein which flap loss was most common. Local swelling, pedicle rotation, tunneling, and dominance of the deep inferior epigastric circulation were potential causes of initial decreased pedicled TRAM perfusion. This analysis adds more objective data to the question of indications and relative strengths between free and pedicled TRAM flaps.

## INTRODUCTION

Since its introduction as a pedicled flap by Hartrampf in 1982, the transverse rectus abdominis myocutaneous (TRAM) flap rapidly became one of the most important flaps in the reconstruction surgeon’s armamentarium.^1^ In breast reconstruction, the TRAM flap is able to provide a soft, ptotic breast mound with excellent volume and reliable blood supply that generally does not require revision over time nor does it need to be monitored for rupture or replaced as is the case with implant reconstruction.^2^

For these and other reasons the TRAM flap remains a popular flap for reconstruction, especially of the breast and chest wall. Multiple modifications on the original design have been proposed to increase the flap perfusion and reliability, decrease donor site morbidity, and widen the demographic for which the flap is appropriate. Grotting’s introduction of the free TRAM in 1989increased the versatility of the flap dramatically, allowing its use beyond the arc of its pedicle and for other applications beyond breast reconstruction.^3^

In breast reconstruction, the free tissue transfer version of the TRAM and its variants have been championed for decreased morbidity with respect to rectus muscle harvest and for its improved contour medially, due to the lack of tunneling during the transfer of tissue.The relative strengths and indications between the pedicled and free muscle flaps have been long debated. It was hypothesized that the larger inferior epigastric vasculature would result in better flap perfusion for a free TRAM as compared to the superior epigastric artery utilized in the pedicled TRAM – an assertion which is still debated today.^3^

This vascular discrepancy infers that an increase in perfusion makes the free TRAM suitable for high risk patients such as diabetics or smokers. Studies have demonstrated similar overall outcomes for pedicled and free versions of the flap in terms of operative time, length of hospital stay, rate of complications, cost, time to return to work, abdominal strength, symmetry and other parameters.^2^^,^^4^^,^^5^ Comparing the blood supply of pedicled versus free flaps can be accomplished with various methods. Subjective clinical evaluations are based on capillary refill of the skin, color of the skin paddle, color of blood on pinprick, as well as temperature.^6^^-^^8^

More objective methods include Doppler ultrasound, microdialysis, implantable doppler devices, and non-invasive measurement of tissue oxygenation.^9^^-^^12^ Another method of flap monitoring via non-invasive imaging techniques is the SPY Elite system (Life Cell Bridgewater, NJ) which uses indocyanine-green (ICG) dye to evaluate vasculature perfusion to the flap including the skin paddle; hence, it can be used to directly evaluate the blood supply to a TRAM flap via the perforating vessels. Previous literature has described the reliability and accuracy of using ICG to evaluate skin perfusion of pedicled or free flaps, including the TRAM.^13^

The SPY Elite system is FDA approved for monitoring and assessing tissue blood flow. Using indocyanine green dye in conjunction with the Novadaq SPY system in a study of 77 free and pedicled TRAM (and including 18 deep inferior epigastric perforator, or DIEP flaps), Loskenet al demonstrated that mean perfusion at the time of harvest, just prior to transfer, was superior in the free flaps as compared to the pedicled.^14^

Whether this increased perfusion persists after transfer intra-operatively and in the early and late post-operative periods was one of the questions in which we were interested. Due to a unique reconstruction completed at our institution, we were afforded the opportunity to simultaneously compare free muscle-sparing and pedicled TRAM flap perfusion quantitatively over time using the SPY Elite. Reconstructive surgeons make further clinical decisions in the weeks to months after autologous tissue reconstructions based on assumptions of the blood flow pattern at this time. Decreased perfusion leads to compromise of the composite tissues brought with the flap, whether skin, muscle, fat and/or bone. 

Proper flap selection for axial based flaps also includes choosing flap harvest on a pedicle or performing a free tissue transfer. An adequately perfused flap is often the only thing that stands between a patient and exposed hardware or important visceral structures. Therefore, knowing what autologous options will provide the most robust blood supply can decrease potential complications related to flap loss. This study capitalized on a unique reconstructive opportunity to directly compare the perfusion of a free muscle-sparing and pedicled TRAM flap side by side in the setting of long term follow up. We hypothesized that through the operative and post-operative period, the free muscle-sparing flap would show earlier and increased perfusion as compared to the matched pedicle flap.

## MATERIALS AND METHODS

**Fig. 1:**
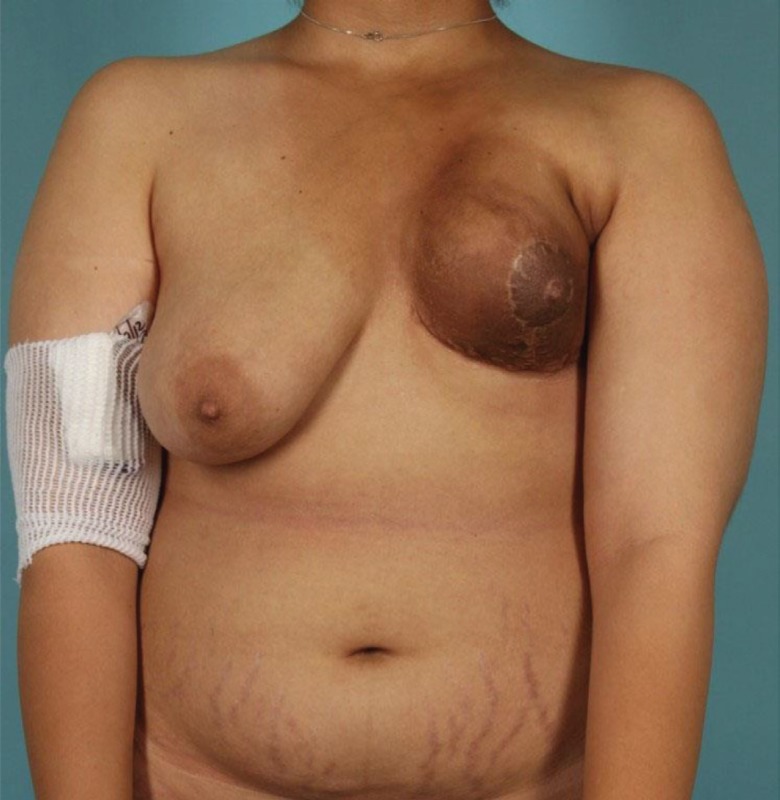
Recurrent left chest well adenocarcinoma after nipple-sparing mastectomy, latissimus dorsi reconstruction, and radiation therapy

**Fig. 2:**
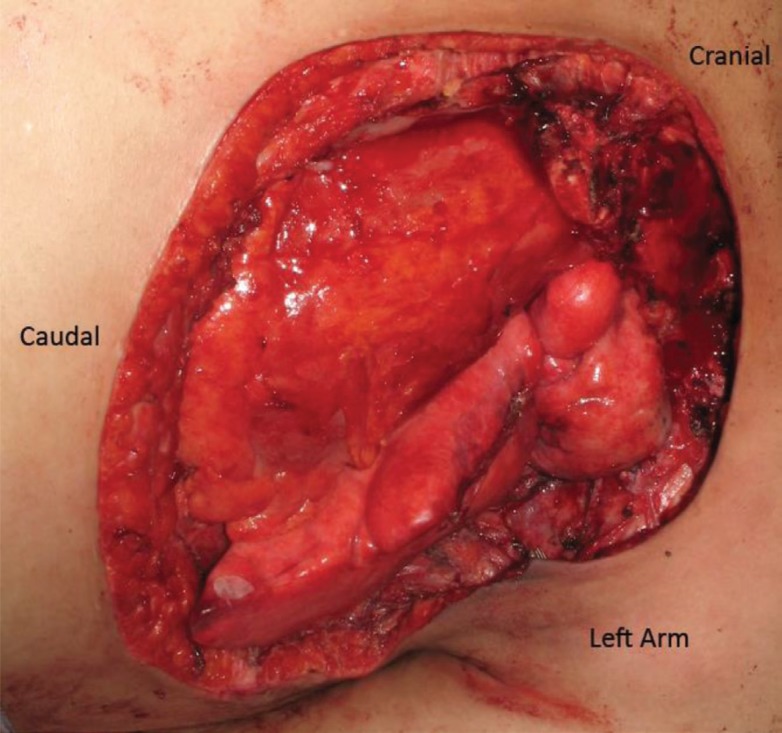
Composite chest wall defect including resection of the left internal mammary vessels

**Fig. 3:**
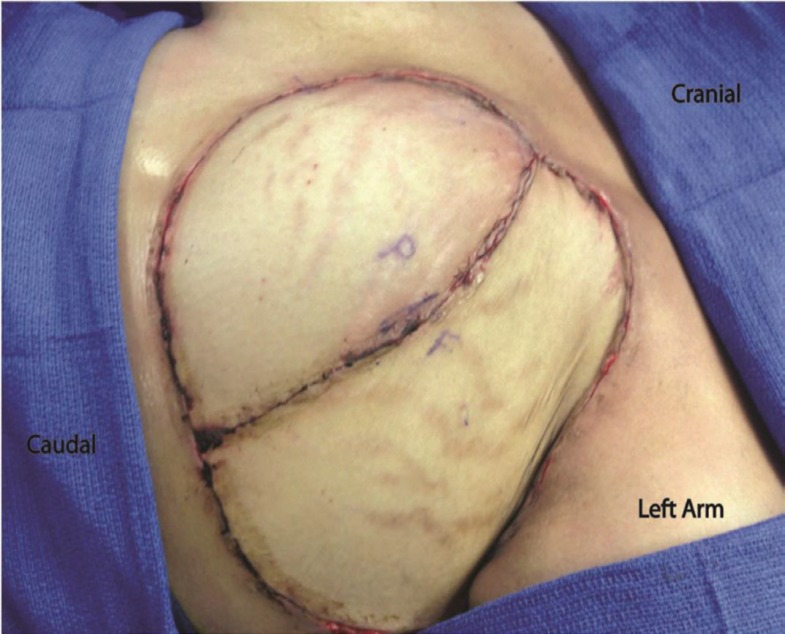
Pedicled and Free TRAM Flaps after inset. Pedicled flap marked with “P”. Free muscle-sparing TRAM marked with “F

The patient was a 27 year old female who presented with a complex poorly differentiated recurrent adenocarcinoma of the left breast with extension through the chest wall (Figure 1). She previously had lumpectomy, radiation, chemotherapy, and reconstruction with latissimus dorsi flap. Composite surgical resection of the left chest wall mass resulted in a significant defect including skin (Figure 2). The defect was not amenable to using a single extended TRAM flap. The lack of suitable recipient vessels due to radiation and extirpation of the left internal mammary artery meant that only one free flap could be harvested, while a pedicle flap would also be needed to complete coverage of the wound. This allowed for the direct *in vivo* comparison of pedicle and free flaps (Figure 3).

**Fig. 4:**
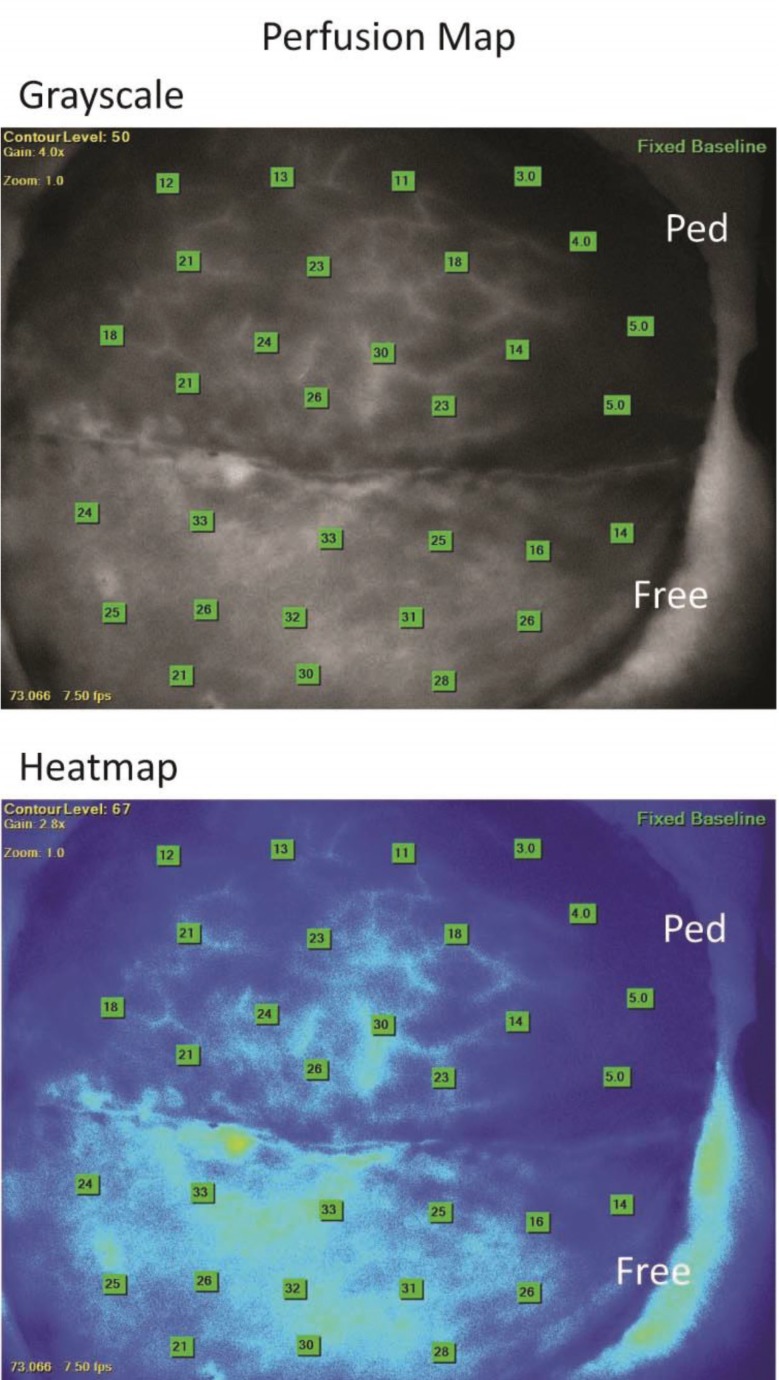
Top: Example of grayscale luminescence. Bottom: False-color heat map. Orientation: Left of page, cranial; Right of page, caudal; top of page, patient left; Bottom of page, patient right. Pedicled TRAM marked as “Ped”. Free muscle-sparing TRAM marked as “Free”. Numbers denote raw luminosity as calculated by SPY Elite system

Level of evidence was diagnostic level IV. The FDA-approved SPY Elite indocyanine-green (ICG, Life Cell, Bridgewater, NJ) angiography perfusion-imaging system was usedto assess free and pedicled TRAM flap perfusion. The SPY system uses a sterilely draped, head-mounted 806nm laser, camera, and infrared filter systemin conjunction with injections of indocyanine green dye(IC-Green; Akorn, Inc., Buffalo Grove, IL) providing real-time,* invivo *images of flap vessels and perfusion. The patient received an intravenous ICG injection via peripheral IV followed by a saline flush. Video, starting at the time of injection and running for 3 minutes, as well as grayscale and colorized heat map photos were captured using the SPY system (Figure 4).

**Fig. 5:**
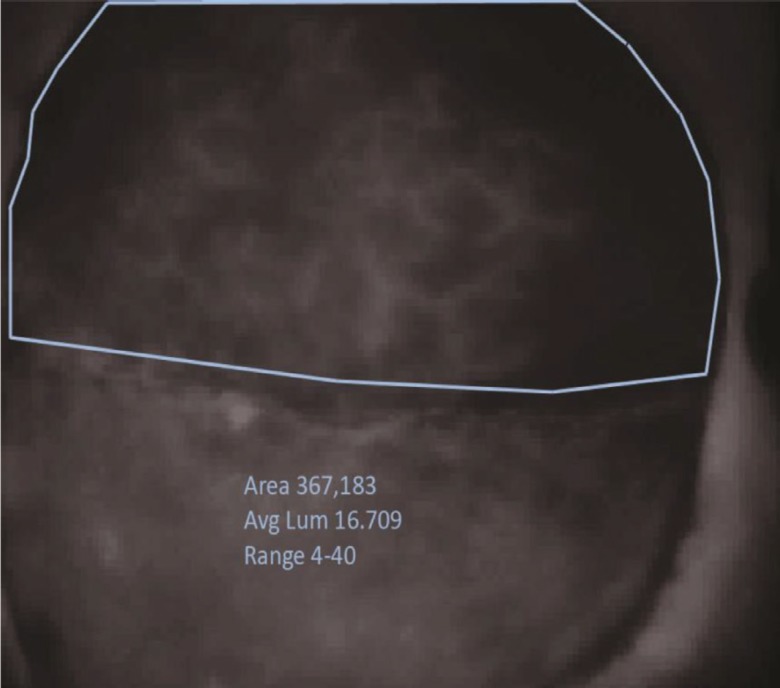
Example of polygon creation to evaluate flap surface area and luminescence

The perfusion analysis videos were retrieved from the SPY elite system using secure USB drive and transferred for data analysis. For all injection videos, a still photo was captured using Media Player Classic freeware (http://mpc-hc.org) at 60 seconds, a time point selected based on signal stability and ICG half life. These photos were analyzed using Image J (NIH, Bethesda, MD). Utilizing the user-controlled polygon tool, a polygon was drawn just within the flap edge or suture line of the pedicled or free muscle-sparing TRAM flaps to avoid artifacts seen at these points (Figure 5). 

The resulting polygon was analyzed for minimum, maximum, and average luminescence based on the Image J calculated area. Each flap at each time point was measured 3 times to yield mean values and standard deviation to account for user variation in polygon placement. *p*-values comparing free and pedicled flaps within each time point were calculated using a Student’s T test with a cut off *p*-value of 0.05 used for statistical significance. This study was conducted under the UC-Irvine Institutional Review Board approved process and guidelines (IRB#2012-9173).

## RESULTS

During the initial reconstruction, the perfusion of the free muscle-sparing and pedicled TRAM flaps were analyzed at 4 time points: pre-division, transposition, pre-inset, and inset. Pre-division is the time prior to flap transposition with pedicled flap isolated on superior epigastric vessels and the free muscle-sparing flap isolated on the deep inferior epigastric artery. Transposition refers to time after transposition of the pedicled flap and microsurgical anastomosis of the free muscle-sparing flap without any fixation with staples or sutures. 

Pre-inset includes de-epithelization, placement of the flaps within defect, and tacking with staples. Inset refers to final fixation with deep dermal and subcuticular sutures of both flaps.

Prior to division of the skeletonized free muscle-sparing flap blood supply or rotation of the pedicled flap, the pedicled flap showed superior perfusion, based on ICG luminosity, with a 54.7% increase over the matched free muscle-sparing flap. After pedicle flap transposition/tunneling and free muscle-sparing flap microsurgical anastomosis, the pedicled TRAM flap showed a 69.3% decrease in perfusion leading to superior free muscle-sparing flap perfusion with a 77.8% higher perfusion as compared to the pedicled flap. 

**Fig. 6:**
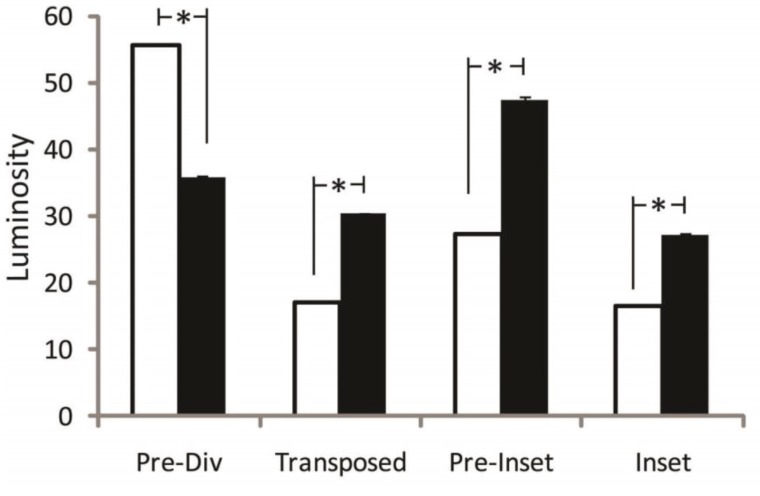
Comparison of free and pedicle flap perfusion at set intra-operative time points. * *p*-value< 0.05

This relative increase in free muscle-sparing flap perfusion compared to the pedicled flap was secondary to a minimal 15.5% decrease in free muscle-sparing flap perfusion with ligation of the deep inferior epigastric vessels and microsurgical anastomosis. This superior free muscle-sparing flap perfusion was maintained during both pre-inset and inset with 73.7% and 64.5% higher luminosity, and thus perfusion, of the free muscle-sparing as compared to the pedicled flap respectively (Figure 6).

Flap perfusion was reassessed at 2 time points post-operatively. The first was on post-operative day 9 during a return to the operating room for pedicle flap debridement and revision due to partial necrosis of the pedicled flap. The second was at 4 months post-operatively for long term analysis of flap perfusion outside of the immediate post-operative period. Both flaps showed significantly increased perfusion throughout the post-operative period. The pedicled flap showed a 4.7 and 5.8 fold increase in perfusion on post-operative day 9 and 4 months post-operatively respectively as compared to its perfusion seen during the initial reconstruction at the final inset time point. 

**Fig. 7:**
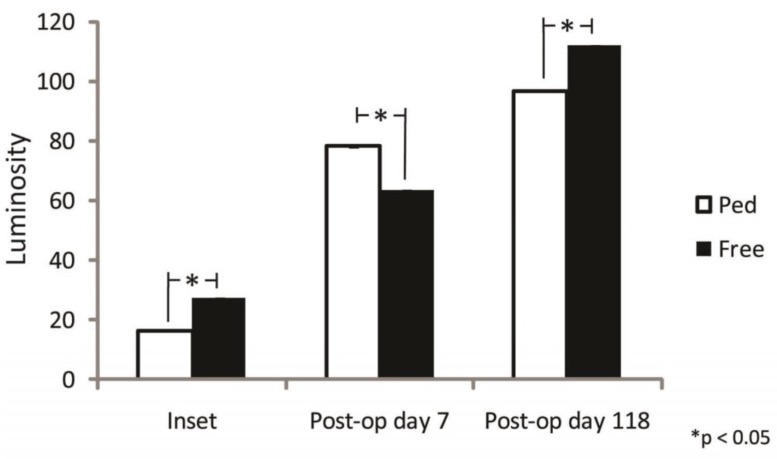
Comparison of post-operative pedicled and free TRAM perfusion. * *p*-value< 0.05

The free muscle-sparing flap similarly showed a 2.3 and 4.1 fold increase in luminosity at these time-points. With this increase in pedicle and free muscle-sparing flap perfusion post-operatively, both flaps surpassed even their pre-division intra-operative perfusions within the first week. On post-operative day 9, the pedicled flap showed a 22.9% greater perfusion as compared to the free muscle-sparing flap after debridement of the distal tip of the pedicled flap secondary to necrosis. This relationship was reversed at 4 months post-operatively with 15.7% higherfree muscle-sparing flap perfusion as compared to the pedicled flap (Figure 7).

## DISCUSSION

Reconstructive surgery dogma has long been that free tissue transfers allow for a safe and reliable reconstructive option for higher risk patients such as those with significant smoking and diabetes history. As compared to a pedicle flap, harvesting a free flap is a more arduous and time-consuming process. This exposes the patient to longer anesthesia times, longer ICU stays, greater healthcare costs and often a greater initial risk of flap loss. In actuality, there is a paucity of data in the literature that has directly compared free and pedicle flap perfusion over an extended time period. 

This study sought to further elucidate the perfusion of free and pedicled flaps both in the initial perioperative time period and with long term analysis. Previous studies have clearly shown that ICG dye with the Spy Elite system can be used to predictably look at the skin perfusion and therefore perforator blood supply of TRAM flaps.^14^ This study capitalized on a unique opportunity to look at the skin/perforator perfusion of a pedicled and free muscle-sparing TRAM flap side by side in the same patient over time. The rectus abdominis muscle flap has long been used as a workhorse flap for breast and chest wall reconstruction and is a valuable resource in the armamentarium of any reconstructive surgeon.^14^^-^^16^

As a Mathes-Nahai Type III muscle flap,^15^^,^^16^ the rectus abdominis is unique in the sheer variability of harvesting options based on desired skin paddle size and vascular supply, namely the superior or deep inferior epigastric artery. Use of the TRAM flap as both a pedicled and free flap in breast and chest wall reconstruction make it a prime candidate for comparison of pedicled and free flap perfusion.

The use of ICG with the Spy Elite system allowed for visual analysis and comparison of perfusion in the operating room between the flaps. We evaluated the flaps just prior to division (after complete dissection of the free muscle-sparing flap vessels), after transposition, and with preliminary and definitive flap inset. The free muscle-sparing flap had greater perfusion after transposition and during inset with a range of 64.5% to 77.8% higher perfusion as compared to the pedicled flap. 

Interestingly, the pedicled TRAM had a 54.7% greater perfusion prior to free muscle-sparing flap division. At first this seemed to act in contrast to the hypothesis. However after careful consideration this may be an expected result. Prior to vessel division, the free muscle-sparing TRAM was dissected to a point where the deep inferior epigastric was only supplying the medial row perforators and the rectus muscle had been partially divided (muscle sparing) to only include the medial row of perforators. 

The pedicle TRAM incorporates all of the medial and lateral row perforators from the superior epigastric artery and does not vertically segment the muscle. Therefore the deep inferior epigastric vessel of the free flap feeds an equally sized skin paddle through fewer vessels. Based on the simplest form of Bernoulli’s principle, the greater total cross sectional area of perforators to the pedicled flap at an equivalent blood pressure should equal greater perfusion. Manipulation of the artery during skeletonization may have lead to arterial vasospasm in the free muscle-sparing TRAM. This would not be present in the pedicled TRAM as there is no direct arterial manipulation.

The free muscle-sparing TRAM had clearly superior perfusion after pedicled flap transposition/tunneling and free flap microsurgical transfer. These data supports that the combination of rotation, tunneling, and resultant edema in the pedicled flap has an immediate effect on flap perfusion with asignificant 69.3% decrease seen in the pedicled flap in this analysis. In contrast, the free muscle-sparing TRAM is oriented and inset in such a way as to avoid kinking of the vessel and pressure on the flap which is supported by a minimal 15.5% decrease in free muscle-sparing flap perfusion with transfer. 

The methods of harvest, transfer, and inset are significantly different between the two flap variations and clearly have a profound effect on vessel dynamics leading ultimately to differences in skin paddle perfusion. Reassessment on postoperative day 9 showed equilibration of pedicled flap perfusion with the caveat that there was an area of ischemic necrosis at the distal area of the pedicle flap that required revision. In contrast, the skin paddle of the free muscle-sparing TRAM flap showed 100 percent viability at all time points. 

This area of flap loss in the pedicle flap was likely secondary to initial flap edema and pedicle rotation as mentioned above. Important to note is that perfusion in all tissue outside of the area of ischemic necrosis had equal perfusion to the free muscle-sparing flap at this time point as measured by fluorescent intensity. While there were small differences seen in perfusion between the two flaps on postoperative days 9 and at 4 months, these differences were deemed clinically insignificant in the face of vastly increased perfusion of both flaps during the first post-operative week. 

The pedicled flap showed a 4.7 and 5.8 fold increased in perfusion on post-operative day 9 and at 4 months as compared to perfusion seen at the final inset during initial reconstruction. The free muscle-sparing flap showed a similar 2.3 and 4.1 fold increase in perfusion at these time points. These increases placed the flaps generally outside of the clinical realm of concern over flap loss. Due to these perfusion increases it is likely that the early vascular insults to the flaps carry more weight in flap viability.

Increased initial free muscle-sparing TRAM perfusion compared to a tunneled, pedicle TRAM allows inset of a larger skin paddle, which would be challenged in a pedicled flap. Local swelling, pedicle rotation and tunneling are potential causes of initial decreased pedicled TRAM perfusion. As would be expected, as the swelling starts to decrease on the 3^rd^postoperative day, the pedicle flap will see stabilization of its perfusion and our results reflected thisas measured on post-operative day 9.

Microvascular free tissue transfer and standard pedicle flap transfer each have their strengths and weaknesses. With a strict comparison of perfusion, this data supports that free tissue transfer has an initial advantage within the first nine days after surgery. Defined in clinical terms, a free tissue transfer is less likely to have partial flap loss likely secondary to less kinking of the vessel with rotation and less external pressure due to tunneling yielding more degrees of freedom for flap inset in the free TRAM. In essence, this infers that free tissue transfer is useful in higher risk patients due to the initial reliability of blood supply and would provide more stable coverage for high risk wounds including exposed hardware or visceral structures. 

This study was able to capitalize on a unique opportunity to compare vascular flow between pedicle and free muscle-sparing TRAM flaps using SPY while generating interesting and thought provoking data regarding perfusion of free and pedicled flaps. Lower perfusion in the free muscle-sparing TRAM at the pre-division time point is likely related to decreased perforators secondary to arterial skeletonization as well as vasospasm from manipulation. Perfusion analysis at the transposition, pre-inset, and inset clearly demonstrate the inherent weakness of the pedicle TRAM causing ischemic insults from tunneling, rotation, edema, and kinking. 

Recovery of the pedicled TRAM flap perfusion outside of the peri-operative period demonstrates that if the flap survives the rotation and tunneling the re-vascularization of the flap from the surrounding tissues is equally as robust. While prospective in nature, this study is limited due to the small sample size. Future studies using side by side pedicled and free flaps in animal models would be required to generalize these conclusions. Until that time, this study advances our knowledge of perfusion dynamics in free and pedicled tissue transfers applicable to the indications and use of these flaps in a multitude of reconstructive applications as well as highlighting the reliability of the Spy system as a tool to analyze flap perfusion.

## Conflict of interest

The authors declare no conflict of interest.
